# Breast cancer biologic and etiologic heterogeneity by young age and menopausal status in the Carolina Breast Cancer Study: a case-control study

**DOI:** 10.1186/s13058-016-0736-y

**Published:** 2016-08-04

**Authors:** Lynn Chollet-Hinton, Carey K. Anders, Chiu-Kit Tse, Mary Beth Bell, Yang Claire Yang, Lisa A. Carey, Andrew F. Olshan, Melissa A. Troester

**Affiliations:** 1 Department of Epidemiology, University of North Carolina, Chapel Hill, NC USA; 2 Lineberger Comprehensive Cancer Center, University of North Carolina, Chapel Hill, NC USA; 3 Division of Hematology-Oncology, Department of Medicine, University of North Carolina, Chapel Hill, NC USA; 4 Carolina Population Center, University of North Carolina, Chapel Hill, NC USA; 5 Department of Sociology, University of North Carolina, Chapel Hill, NC USA; 6 Department of Medicine, School of Medicine, University of North Carolina, Chapel Hill, NC USA; 7 Department of Pathology and Laboratory Medicine, University of North Carolina, Chapel Hill, NC USA

**Keywords:** Breast cancer, Epidemiology, Age, Risk factors, Tumor characteristics

## Abstract

**Background:**

Young-onset breast cancer (<40 years) is associated with worse prognosis and higher mortality. Breast cancer risk factors may contribute to distinct tumor biology and distinct age at onset, but understanding of these relationships has been hampered by limited representation of young women in epidemiologic studies and may be confounded by menopausal status.

**Methods:**

We examined tumor characteristics and epidemiologic risk factors associated with premenopausal women’s and young women’s breast cancer in phases I–III of the Carolina Breast Cancer Study (5309 cases, 2022 control subjects). Unconditional logistic regression was used to assess heterogeneity by age (<40 vs. ≥40 years) and menopausal status.

**Results:**

In both premenopausal and postmenopausal strata, younger women had more aggressive disease, including higher stage, hormone receptor-negative, disease as well as increased frequency of basal-like subtypes, lymph node positivity, and larger tumors. Higher waist-to-hip ratio was associated with reduced breast cancer risk among young women but with elevated risk among older women. Parity was associated with increased risk among young women and reduced risk among older women, while breastfeeding was more strongly protective for young women. Longer time since last birth was protective for older women but not for young women. In comparison, when we stratified by age, menopausal status was not associated with distinct risk factor or tumor characteristic profiles, except for progesterone receptor status, which was more commonly positive among premenopausal women.

**Conclusions:**

Age is a key predictor of breast cancer biologic and etiologic heterogeneity and may be a stronger determinant of heterogeneity than menopausal status. Young women’s breast cancer appears to be etiologically and biologically distinct from that among older women.

**Electronic supplementary material:**

The online version of this article (doi:10.1186/s13058-016-0736-y) contains supplementary material, which is available to authorized users.

## Background

Breast cancer among young women (<40 years of age) is associated with more proliferative disease, worse prognosis, and higher mortality than disease among older women (≥40 years) [1–6]. Young women have larger tumors, higher-grade disease, hormone receptor negativity, and lymph node positivity, which may contribute to poorer disease outcomes [4–10]. Differences in the distribution of breast cancer risk factors (particularly race, body size, and reproductive exposures) among younger vs. older women may also contribute to differences in tumor characteristics by age [7, 11–14]. However, findings regarding associations between breast cancer risk factors and age at diagnosis have been inconsistent. Resolution of previous inconsistencies has been impeded by limited representation of young women in most studies [7, 13–15], as less than 7 % of all breast cancers in the United States are diagnosed among women <40 years of age [5, 16] and by differences in defining “young” women, with some studies equating young age with premenopausal status and others using a variety of “young” age cutoffs [13–15, 17–20]. While age and menopausal status are strongly associated, these variables may have independent effects on cancer development [6].

The present study was conducted within the Carolina Breast Cancer Study (CBCS), a large, population-based case-control study of breast cancer biology and epidemiology enriched for breast cancers occurring among young women. In our analysis, we had two objectives: (1) to identify breast tumor characteristics and epidemiologic risk factors associated with young vs. older women’s breast cancer in the CBCS and (2) to assess heterogeneity in these associations independently by age (<40 years vs. ≥40 years) and menopausal status (pre- vs. postmenopausal).

## Methods

### Study population

The CBCS is a population-based case-control study conducted in 24 (phases I and II) and 44 (phase III) counties of central and eastern North Carolina. Women were eligible for inclusion if they were 20–74 years of age at the time of diagnosis (cases) or study recruitment (control subjects). As described previously [17, 21–23], the CBCS researchers collected extensive clinical, molecular, and epidemiologic data for in situ (phase II) and invasive (phases I–III) breast cancer cases diagnosed from 1993 to 1996 (phase I), 1996 to 2001 (phase II), and 2008 to 2013 (phase III). Cases were identified through rapid case ascertainment by the North Carolina Central Cancer Registry; control subjects were recruited (for phases I and II only) through North Carolina Department of Motor Vehicles records and Health Care Financing Administration records for Medicare enrollment. Phase III was conducted in 44 counties and recruited only patients with invasive breast cancer. In all phases, randomized recruitment was used to oversample younger (<50 years) and African American cases as well as to frequency-match control subjects to cases by age (<50 years vs. ≥50 years), 5-year age group, and self-reported race (African American vs. non-African American) [21]. The response rates for cases and control subjects in phases I and II have been reported previously [24]. For phase III cases, the overall contact rate (contacted/eligible) was 95.5 %, the cooperation rate (enrolled/contacted) was 75.8 %, and the overall response rate (product of contact and cooperation rates) was 72.0 %. The present analysis included all cases (*n* = 5309) from phases I–III for case-case analyses as well as phases I and II cases (*n* = 2311) and control subjects (*n* = 2022) for case-control analyses. All study protocols were approved by the University of North Carolina (UNC) School of Medicine Institutional Review Board, and all participants gave their written informed consent.

### Data collection

Breast tumor tissue and medical records were obtained from local hospitals for all in situ and invasive breast cancer cases. Stage at diagnosis (in situ, stages I–IV), lymph node status (positive vs. negative for metastasis), estimated tumor size (>2 cm, ≤2 cm), nuclear grade (marked, slight/moderate pleomorphism), and histologic grade (poorly, moderately/well-differentiated) were obtained from medical record reviews, although nuclear and histologic grade data were unavailable for all phase II cases. Immunohistochemistry (IHC) assays conducted at the UNC Immunohistochemistry Core Laboratory were completed to define human epidermal growth factor receptor 2 (HER2) status (positive defined as ≥10 % cells staining vs. negative) [24]. Estrogen receptor (ER) and progesterone receptor (PR) status information was obtained from medical records when available (88 %) or by IHC at UNC (12 %), with ER/PR positivity defined as ≥5–10 % cells with nuclear staining [25]. A 10 % random sample of cases showed high agreement between ER status obtained from medical records and IHC assays at UNC (*k*-statistic = 0.62, concordance = 81 %) [25]. Breast cancer subtype was defined on the basis of ER, PR, HER1, HER2, and cytokeratin (CK) 5/6 positivity as previously described [25]: basal-like (ER^−^, PR^−^, HER2^−^, HER1^+^, and/or CK 5/6^+^), luminal (ER^+^ and/or PR^+^), HER2^+^/ER^−^ (ER^−^, PR^−^, HER2^+^), and unclassified (negative for all five markers). Collection of molecular subtype data is ongoing for phase III cases; only phases I and II cases were included in analyses of these characteristics.

In-home interviews were conducted by study nurses for all cases and control subjects [17]. Participants were asked questions regarding their reproductive and medical histories as well as exogenous hormone exposures. Nurses also measured body weight, height, waist circumference, and hip circumference during the interview. All breast cancer risk factors were categorized as previously reported [17]. Briefly, menopausal status was defined as pre- or postmenopausal on the basis of self-reported cessation of menstruation for women over age 50 years; women under age 50 years were defined as premenopausal unless they had reported undergoing menopause-related cessation of menstruation, bilateral oophorectomy, or ovarian irradiation. Body mass index (BMI) was defined as body weight/height ratio in kilograms per square meter using categories of the National Heart, Lung, and Blood Institute (normal/underweight <25 kg/m^2^, overweight 25.0–29.9 kg/m^2^, and obese ≥30 kg/m^2^) [26]. Waist-to-hip ratio (WHR) was calculated as the waist/hip circumference ratio in centimeters and categorized as <0.77 cm, 0.77–0.83 cm, and ≥0.84 cm. Classifications for other risk factors were consistent with previous CBCS analyses [17]: age at menarche (<13 years, ≥13 years), parity (nulliparous, 1–2 births, ≥3 births), age at first live birth (<26 years, ≥26 years), history of breastfeeding (never, ever), lifetime duration of breastfeeding (never, >0–3 months, ≥4 months), and oral contraceptive use (never, ever).

### Statistical analysis

To examine the baseline exposure distributions, descriptive analyses examining the distribution of breast cancer risk factors among all control subjects were conducted. For case-case analyses of tumor characteristics, unconditional logistic regression was used to calculate ORs and 95 % CIs. Age and menopausal status are strongly correlated but may influence breast cancer etiology through distinct mechanisms. To estimate age effects independent of menopausal status, case-case analyses were restricted to premenopausal cases (*n* = 2373). Similarly, to consider the effects of menopause independent of age, case-case analyses were conducted among women ≥40 years of age. However, postmenopausal women ≥40 years of age represent a wide range of ages (40–74 years), and associations estimated within this group are susceptible to residual confounding by age. Therefore, we conducted a sensitivity analysis estimating case-case associations for tumor characteristics among pre- vs. postmenopausal women aged 40–49 years and 40–59 years of age. Analyses were conducted among all women and stratified by race (African American and non-African American) to examine effect measure modification by race.

Case-control analyses were conducted for breast cancer risk factors, stratifying by age at diagnosis or menopausal status. Unconditional logistic regression, with an offset term to account for the sampling probabilities of cases and control subjects, was used to estimate case-control ORs and 95 % CIs for each risk factor. Young (<40 years) vs. older (≥40 years) groups were evaluated among premenopausal women and pre- vs. postmenopausal status was evaluated among women ≥40 years. All analyses were adjusted for age (5-year age categories) and race (African American vs. non-African American). Similar to the case-case analyses described above, we restricted our analyses of risk factor associations by menopausal status to pre- and postmenopausal women aged 40–59 years to address residual confounding by age and to control for nonpositivity (i.e., the lack of premenopausal women above age 59 years). Heterogeneity in risk factor associations by age or menopausal status was evaluated by conducting likelihood ratio tests in which the estimated log-likelihood of adjusted models was compared with that of the adjusted model including a multiplicative interaction term for age (or menopausal status) and the corresponding risk factor. Statistically significant heterogeneity was defined with α = 0.1. To explore racial differences in etiologic heterogeneity by age and menopausal status, case-control analyses were further stratified by race. Models for age at first live birth, history of breastfeeding, and lifetime breastfeeding duration were restricted to parous women. Statistical significance was defined with α = 0.05. All analyses were performed using SAS version 9.3 software (SAS Institute, Cary, NC, USA).

## Results

To identify possible confounders of the risk factor and age-at-diagnosis association, we examined the distribution of breast cancer risk factors among young (<40 years) vs. older (≥40 years) control subjects (Table 1). Although the majority of control subjects were white (59.3 % vs. 40.7 % for African American and other race combined), young control subjects were more likely to be African American or of another race. Young control subjects had lower BMI and WHR, earlier menarche, lower parity, older age at first birth, greater history of ever breastfeeding, longer duration of breastfeeding, and higher oral contraceptive use than older control subjects. Risk factors that showed differential distributions among control subjects, overall and by race (Additional file 1: Table S1), were included in multivariable models.

**Table 1:** Characteristics of control subjects by age in Carolina Breast Cancer Study phases I and II (*n* = 2022)

Risk factor	<40 years, *n* (%)	≥40 years, *n* (%)
Mean age (±SD)	35.3 (±3.2)	54.8 (±9.9)
Race		
White	120 (52.0)	1080 (60.3)
African American	104 (45.0)	684 (38.2)
Other	7 (3.0)	27 (1.5)
BMI		
< 25 kg/m^2^ (normal/underweight)	97 (42.0)	652 (37.0)
25–29.9 kg/m^2^ (overweight)	60 (26.0)	541 (30.7)
≥ 30 kg/m^2^ (obese)	74 (32.0)	570 (32.3)
Missing		28
WHR		
< 0.77	92 (41.1)	523 (29.6)
0.77–0.83	71 (31.7)	575 (32.5)
≥ 0.84	61 (27.2)	671 (37.9)
Missing	7	22
Parity		
Nulliparous	50 (21.7)	180 (10.1)
1–2 births	138 (59.7)	875 (48.9)
≥ 3 births	43 (18.6)	736 (41.1)
History of breastfeeding^a^		
Never	83 (45.9)	906 (56.4)
Ever	98 (54.1)	700 (43.6)
Lifetime breastfeeding duration^a^		
Never	92 (50.8)	925 (57.7)
> 0–3 months	27 (14.9)	225 (14.0)
≥ 4 months	62 (34.3)	453 (28.3)
Missing		3
Time since last term pregnancy^a^		
< 10 years	124 (68.5)	82 (5.1)
10–19 years	53 (29.3)	397 (24.7)
≥ 20 years	4 (2.2)	1126 (70.2)
Age at first live birth^a^		
< 26 years	111 (61.3)	1235 (76.9)
≥ 26 years	70 (38.7)	371 (23.1)
Age at last live birth^a^		
< 30 years	109 (60.2)	976 (60.8)
≥ 30 years	72 (39.8)	629 (39.2)
Missing		1
Age at menarche		
< 13 years	117 (50.9)	825 (46.2)
≥ 13 years	113 (49.1)	959 (53.8)
Missing	1	7
Oral contraceptive use		
Never	34 (14.7)	694 (39.0)
Ever	197 (85.3)	1084 (61.0)
Missing		13

*BMI* body mass index, *WHR* waist-to-hip ratio

^a^Among parous women

### Tumor characteristic and risk factor associations according to age at diagnosis

Tumor characteristics for in situ and invasive cases were examined together in this study, as sensitivity analyses did not reveal any difference in results when excluding in situ cases (results not shown) nor any difference in the prevalence of in situ cases by age (Table 2). In logistic regression analyses of the relationship between young age at diagnosis and tumor characteristics, young women had more aggressive tumors (Table 2); specifically, young women’s tumors were more likely to be higher stage and basal-like breast cancers. Young women also had significantly more ER and PR negative disease with a greater frequency of marked pleomorphism, positive lymph nodes, and larger tumor size. Although not significantly different, HER2 positivity and more poorly differentiated histologic grade were more common among young women.

**Table 2:** Case-case ORs of tumor characteristics by age among premenopausal cases in Carolina Breast Cancer Study phases I–III (*n* = 2373)

	Age <40 years		Age ≥40 years (reference)
Tumor characteristic	*n* (%)	OR (95 % CI)	*n* (%)
Mean age (±SD)	34.8 (±3.7)		45.4 (±3.5)
Stage			
In situ	35 (5.4)	0.98 (0.65–1.49)	125 (7.5)
Stage I	163 (25.3)	1.0	573 (34.5)
Stage II	340 (52.7)	1.74 (1.40–2.16)	687 (41.4)
Stage III	84 (13.0)	1.34 (0.99–1.82)	220 (13.3)
Stage IV	23 (3.6)	1.47 (0.88–2.46)	55 (3.3)
Missing	19		51
Subtype^a^			
Luminal	106 (52.0)	1.0	283 (65.2)
Basal-like	51 (25.0)	1.87 (1.22–2.84)	73 (16.8)
HER2	18 (8.8)	1.72 (0.91–3.23)	28 (6.5)
Unclassified	29 (14.2)	1.55 (0.93–2.58)	50 (11.5)
Missing	109		286
ER status			
Negative	270 (44.9)	1.0	515 (33.4)
Positive	332 (55.1)	0.62 (0.51–0.75)	1028 (66.6)
Missing^b^	62		168
PR status			
Negative	302 (53.6)	1.0	544 (37.6)
Positive	261 (46.4)	0.52 (0.43–0.63)	903 (62.4)
Missing^b^	101		264
HER2 status^a^			
Negative	194 (77.0)	1.0	447 (82.3)
Positive	58 (23.0)	1.39 (0.96–2.01)	96 (17.7)
Missing	61		177
Histologic grade^c^			
Well-differentiated/moderate differentiation	89 (21.3)	1.0	268 (25.6)
Poor differentiation	328 (78.7)	1.27 (0.97–1.66)	779 (74.4)
Missing^b^	82		238
Nuclear grade^c^			
Slight/moderate pleomorphism	174 (41.2)	1.0	557 (52.2)
Marked pleomorphism	248 (58.8)	1.55 (1.24–1.95)	511 (47.8)
Missing^c^	77		217
Node status			
Negative	329 (53.4)	1.0	907 (58.4)
Positive	287 (46.6)	1.23 (1.02–1.48)	645 (41.6)
Missing^b^	48		159
Tumor size			
≤ 2 cm	233 (38.4)	1.0	749 (48.8)
> 2 cm	373 (61.6)	1.53 (1.26–1.85)	786 (51.2)
Missing^b^	58		176

*ER* estrogen receptor, *HER2* human epidermal growth factor receptor 2, *PR* progesterone receptor

^a^Excludes phase III cases

^b^Missing data due to ongoing data collection for phase III cases

^c^Excludes phase II cases

To evaluate whether young- and older-onset breast cancers are etiologically distinct, we estimated associations with risk factors stratified by age (Table 3). These analyses were restricted to premenopausal women to avoid confounding by menopausal status. Age appeared to modify associations for several risk factors. Ever-breastfeeding and a lifetime breastfeeding duration ≥4 months were associated with reduced risk of young-onset (<40 years of age) breast cancer and no change in risk of older-onset (≥40 years) disease. Likelihood ratio tests showed significant effect measure modification by age for both ever-breastfeeding (interaction *p* = 0.003) and duration of breastfeeding (interaction *p* = 0.04). Parity and longer time since last term pregnancy (10–19 years) were protective among older women and had either null or weakly increased risk for young women, although this heterogeneity was not statistically significant. High WHR was protective among younger cases and either null (BMI) or associated with elevated risk (WHR) among older women. Oral contraceptive use was more strongly, but not significantly, associated with risk among younger women, while associations with BMI, age at first birth, age at last birth, and age at menarche were similar between young and older premenopausal cases. Thus, patterns of risk factor associations differed substantially between young and older premenopausal women.

**Table 3:** Case-control ORs of breast cancer risk factors by age among premenopausal women in Carolina Breast Cancer Study phases I and II (*n* = 1904)

	Age <40 years			Age ≥40 years			Test for heterogeneity
Risk factor	Control subjects, *n* (%)	Cases, *n* (%)	OR (95 % CI)^a^	Control subjects, *n* (%)	Cases, *n* (%)	OR (95 % CI)^a^	χ^2^ test, *df* (*p* value)^b^
BMI, kg/m^2^							
< 25.0	93 (41.9)	154 (49.2)	1.0	255 (40.0)	316 (44.3)	1.0	0.13, 2 (0.9)
25–29.9	58 (26.1)	71 (22.7)	0.72 (0.45–1.13)	184 (28.9)	181 (25.4)	0.87 (0.65–1.14)	
≥ 30.0	71 (32.0)	88 (28.1)	0.73 (0.47–1.13)	198 (31.1)	217 (30.4)	0.98 (0.74–1.29)	
Missing				12	6		
WHR							
< 0.77	89 (41.4)	135 (44.3)	1.0	235 (36.8)	231 (32.4)	1.0	2.47, 2 (0.3)
0.77–0.83	68 (31.6)	98 (32.1)	0.94 (0.61–1.43)	209 (32.7)	245 (34.4)	1.42 (1.08–1.86)	
≥ 0.84	58 (27.0)	72 (23.6)	0.86 (0.54–1.36)	195 (30.5)	237 (33.2)	1.58 (1.18–2.11)	
Missing	7	8		10	7		
Parity							
Nulliparous	49 (22.1)	70 (22.4)	1.0	76 (11.7)	106 (14.7)	1.0	3.55, 2 (0.2)
1–2 births	133 (59.9)	180 (57.5)	1.05 (0.67–1.63)	383 (59.0)	420 (58.3)	0.82 (0.58–1.14)	
≥ 3 births	40 (18.0)	63 (20.1)	1.29 (0.74–2.27)	190 (29.3)	194 (26.9)	0.81 (0.55–1.18)	
History of breastfeeding^c^							
Never	77 (44.5)	145 (60.2)	1.0	343 (60.2)	370 (60.6)	1.0	9.01, 1 (0.003)
Ever	96 (55.5)	96 (39.8)	0.48 (0.31–0.75)	227 (39.8)	241 (39.4)	0.90 (0.70–1.16)	
Lifetime breastfeeding duration^c^							
Never	86 (49.7)	148 (61.4)	1.0	346 (60.7)	378 (61.9)	1.0	6.36, 2 (0.04)
> 0–3 months	25 (14.5)	33 (13.7)	0.79 (0.43–1.45)	69 (12.1)	65 (10.6)	0.86 (0.58–1.25)	
≥ 4 months	62 (35.8)	60 (24.9)	0.50 (0.30–0.81)	155 (27.2)	168 (27.5)	0.87 (0.65–1.15)	
Time since last term pregnancy^c^							
< 10 years	119 (68.8)	169 (70.1)	1.0	76 (13.3)	108 (17.7)	1.0	4.44, 2 (0.1)
10–19 years	50 (28.9)	66 (27.4)	1.02 (0.64–1.63)	302 (53.0)	283 (46.3)	0.71 (0.50–1.01)	
≥ 20 years	4 (2.3)	6 (2.5)	1.14 (0.30–4.27)	192 (33.7)	220 (36.0)	0.96 (0.64–1.43)	
Age at first live birth^c^							
< 26 years	103 (59.5)	146 (60.6)	1.0	386 (67.7)	414 (67.8)	1.0	0.00, 1 (1.0)
≥ 26 years	70 (40.5)	95 (39.4)	1.03 (0.68–1.58)	184 (32.3)	197 (32.2)	0.93 (0.71–1.20)	
Age at last live birth^c^							
< 30 years	102 (59.0)	148 (61.4)	1.0	344 (60.4)	362 (59.2)	1.0	0.01, 1 (0.9)
≥ 30 years	71 (41.0)	93 (38.6)	1.01 (0.67–1.54)	226 (35.6)	249 (40.8)	0.99 (0.78–1.26)	
Age at menarche							
< 13 years	111 (50.2)	170 (54.3)	1.0	323 (49.8)	393 (54.6)	1.0	0.01, 1 (0.9)
≥ 13 years	110 (49.8)	143 (45.7)	0.85 (0.59–1.20)	326 (50.2)	327 (45.4)	0.83 (0.66–1.03)	
Missing	1						
Oral contraceptive use							
Never	33 (14.9)	36 (11.5)	1.0	112 (17.3)	125 (17.4)	1.0	0.67, 1 (0.4)
Ever	189 (85.1)	276 (88.5)	1.32 (0.78–2.22)	534 (82.7)	593 (82.6)	0.93 (0.70–1.25)	
Missing		1		3	2		

*BMI* body mass index, *WHR* waist-to-hip ratio

^a^Adjusted for matching factors race and age

^b^Likelihood ratio tests were performed to assess age-related heterogeneity in risk factor associations by comparing the estimated log-likelihood of adjusted models with that of the adjusted model including a multiplicative interaction term for age and the corresponding risk factor (e.g., BMI × age). Statistically significant heterogeneity by age was defined with α = 0.1

^c^Among parous women

### Tumor characteristic and risk factor associations by menopausal status

Relative to the age patterns described above, associations with menopausal status were attenuated. After restricting to cases ≥40 years of age, we assessed the association between menopausal status and tumor characteristics. In crude analyses, premenopausal status appeared to be associated with poor-prognosis tumor characteristics similar to those observed when we compared young and older premenopausal women. Premenopausal cases were significantly less likely to have in situ disease and ER positivity and more likely than postmenopausal women to have stage II, III, or IV disease; higher histologic and nuclear grade; lymph node positivity; and greater tumor size (Additional file 2: Table S2). However, after adjusting for age and/or restricting the age range of women to control for nonpositivity (i.e., the lack of premenopausal women above age 59 years), we observed that few changes persisted. Among women aged 40–49 years, only PR status and nuclear grade showed differences by menopausal status; premenopausal women had higher PR positivity and less marked pleomorphism. Among women aged 40–59 years, premenopausal women had a greater likelihood of stage II and PR^+^ disease as well as larger tumors than postmenopausal women (Additional file 2: Table S2); however, strata for premenopausal women over age 50 years were very sparse, leading to some instability of estimates. These results suggest that, while menopausal status may be associated with some tumor characteristics, associations are weaker than those by age.

Similarly, stratification on menopausal status showed limited evidence of etiologic heterogeneity (Additional file 3: Table S3). Only age at last birth showed significant modification by menopausal status (*p* = 0.01), in that age at last birth ≥30 years was not associated with increased risk among premenopausal women but did increase risk among postmenopausal women. No other risk factors were differentially associated with pre- or postmenopausal breast cancer after adjusting for age and race.

### Racial differences in tumor characteristics and risk factor associations by age and menopausal status

In race-stratified analyses, associations between young age and tumor characteristics were more precise among white women but were similar in direction (results not shown). Only HER2 positivity, lymph node positivity, and histologic grade showed a suggestion of heterogeneity by race: Young age was associated with increased HER2 positivity (OR 1.72, 95 % CI 1.08–2.72) and increased lymph node positivity (OR 1.35, 95 % CI 1.04–1.76) among premenopausal white women but not African American women (HER2 OR 1.03, 95 % CI 0.56–1.92; lymph node OR 1.07, 95 % CI 0.82–1.40). Additionally, among white women, younger cases had a higher prevalence of poorly differentiated histologic grade (OR 1.63, 95 % CI 1.13–2.36); this association was qualitatively different among African American women (OR 0.86, 95 % CI 0.57–1.30). No strong heterogeneity by race was observed in the associations between tumor characteristics and menopausal status after controlling for age at diagnosis (results not shown).

Likewise, little heterogeneity was observed by race for associations between breast cancer risk factors and menopausal status. With regard to breast cancer risk factors, higher WHR increased risk among African American women regardless of age, while age appeared to modify the association between WHR and risk in white women (results not shown). History of breastfeeding and longer breastfeeding duration strongly reduced risk among both young and older African American women (history of breastfeeding: young OR 0.50, 95 % CI 0.25–1.00; older OR 0.59, 95 % CI 0.38–0.92; breastfeeding duration: young OR 0.47, 95 % CI 0.20–1.11; older OR 0.56, 95 % CI 0.32–0.98), while the protective effect of breastfeeding in white women appeared to be restricted to young women. No other risk factors were associated with breast cancer risk in race- and age-stratified analyses.

## Discussion

Among premenopausal women in the CBCS, early-onset breast cancers (<40 years) are more aggressive than those among older women. Young women had more advanced stage disease at diagnosis, larger tumors, more hormone receptor-negative disease, marked pleomorphism, and lymph node positivity than older women. We also found that younger cases were more likely to be nonwhite and to have reduced adiposity (lower WHRs) than older cases. We found evidence for the dual effects of parity on risk, similar to other previous studies [14, 27–29]; namely, breast cancer risk was associated with higher parity among young women but not older women. Additionally, greater time since last birth was significantly protective for older women but not young women. Both history of breastfeeding and increased breastfeeding duration significantly reduced the risk of breast cancer among young but not older women, underscoring the importance of breastfeeding in mitigating parity-associated risk among young women [7, 17, 30, 31]. Together these results suggest a unique pattern of risk factors for young women’s breast cancer.

This heterogeneity in the prevalence of aggressive tumor characteristics by age at diagnosis has been well-documented [5–10, 32], and our work supports the hypothesis that young women’s breast cancer may be biologically and/or clinically distinct from disease in older women. Patterns for PR were interesting, first showing that younger women were significantly more likely to have PR negative disease, a finding that is well-established in the literature [5–7, 9]. This is in contrast to our finding that, among older women, premenopausal cases had increased PR positivity compared with postmenopausal cases, even after accounting for differences in age. Talley et al. [33] found a similar suggestion of increased PR positivity in premenopausal (compared with postmenopausal) women, although the association was not statistically significant. The complex effects on PR expression are difficult to interpret and may reflect a combination of etiologic and progression events; young women may be more likely to get receptor negative disease, but older women developing PR^+^ disease may lose receptor expression as menopause ensues. Further work examining PR positivity in relation to age and menopausal status is warranted to replicate these findings. Moreover, many of the associations we detected may reflect a complex combination of etiologic and progression differences. Previous epidemiologic studies have identified heterogeneity in the associations between breast cancer risk factors and risk of distinct tumor subtypes. For example, reproductive and body size exposures are differentially associated with luminal and basal-like tumors [17, 34]. Breastfeeding reduces risk of ER^−^ breast cancer and in our study is associated with young women’s breast cancer. It is possible that patterns of tumor characteristics by age, both in our present study and in other previous studies, reflect these underlying etiologic assumptions.

Young age and premenopausal status have often been used almost interchangeably to define women with early-onset breast cancer; however, we observed that these factors may be better considered as separate factors. While young vs. older age at onset produced patterns similar to those of pre- vs. postmenopausal status, nearly all menopause associations (with the exception of PR status and nuclear grade) became null after restricting on age, suggesting that heterogeneity in breast cancer characteristics by menopausal status was driven by differences in age. Previous studies have also suggested age-confounding of menopause etiologic associations; for example, Lee et al. [35] reported significant differences in the association between dietary exposures and pre- vs. postmenopausal breast cancer that were then found to be attributable to age differences across menopausal status. Given the strong association between age and menopausal status, examining the independent contributions of these factors on breast cancer risk is challenging. In our study, adjusting for age or menopausal status as confounders in regression models was hampered by nonpositivity, as few women <40 years old were postmenopausal in the CBCS. We restricted age comparisons to premenopausal women and menopausal comparisons to older women, thereby estimating the relative contributions of age and menopausal status independently and avoiding nonpositivity issues in our analyses. While analyses restricted on age or menopausal status may limit generalizability (i.e., the results for pre- vs. postmenopausal comparisons may not apply to women with very early menopause before age 40), increased internal validity helps to dissect the relative importance of age and menopausal status in etiology.

We observed limited heterogeneity by race in our analyses, suggesting that young women’s breast cancers may have similar etiologies and biological characteristics regardless of race. However, African American women have higher breast cancer incidence prior to age 40 years [32, 36–38], making young women’s breast cancer particularly relevant. Considering tumor characteristics, it is well-established that poor prognostic features are more prevalent among young and African American women [5, 8, 25], but other work in the CBCS and other study populations has shown that age-associated tumor characteristics are similar by race [6, 8]. Using a subset of the included data presented here, Furberg et al. [8] previously reported that young (<40 years) African American and white women differed only by the prevalence of ER and PR positivity. Considering breast cancer risk factors, only body size (WHR) and breastfeeding were differentially associated with age at onset among African American and white women. Other studies have observed heterogeneity in breast cancer risk by age and race according to parity, oral contraceptive use, and age at menarche [39, 40], associations that we did not observe in our study.

Our results should be interpreted in light of some limitations. Analyses of tumor subtype, HER2 status, and histologic/nuclear grade were limited by missing data for some tumors. Data for tumor grade were unavailable for phase II cases, and data collection for HER2 status is currently ongoing for phase III of the CBCS. In prior work considering the association between HER2 and age at diagnosis, researchers reported mixed findings, although recent studies have suggested increased HER2 positivity among young women [4, 10, 32]. We observed a higher prevalence, though not significantly, of HER2^+^ tumors among young women. Additionally, in our analyses of ER and PR positivity, we used 5–10 % nuclear staining to define hormone receptor positivity, consistent with clinical standards during the study period. However, this cutpoint differs from current guidelines issued by the American Society of Clinical Oncology/College of American Pathologists, which recommend a 1 % positivity cutpoint [41]. Recent findings suggest that a 10 % cutpoint was preferable for identifying intrinsic subtypes [42], suggesting that the impact of clinical thresholds on interpretation of epidemiologic findings remains uncertain. Finally, screen-detected breast cancers tend to be less advanced than those that are self-detected or clinically detected [43, 44], and young women <40 years of age do not typically receive mammographic screening. Screening differences may account for some of the observed heterogeneity in tumor aggressiveness by age, but they would be unlikely to account for differences in etiology.

## Conclusions

Our findings suggest that young women’s breast cancer is biologically and possibly etiologically distinct from breast cancers arising in older women. Age appears to be a key driver for breast cancer heterogeneity that is at least as important, or perhaps even more important, than the effects of menopausal status. Clarifying the etiologic and biological features of young women’s breast cancer is important for identifying modifiable targets for prevention of aggressive, early-onset breast cancers.

## Abbreviations

BMI, body mass index; CBCS, Carolina Breast Cancer Study; CK, cytokeratin; ER, estrogen receptor; HER2, human epidermal growth factor receptor 2; IHC, immunohistochemistry; PR, progesterone receptor; WHR, waist-to-hip ratio

## Additional files

Additional file 1: Table S1. Characteristics of control subjects by age and race in the Carolina Breast Cancer Study phases I and II (*n* = 2022). (DOC 61 kb) Additional file 2: Table S2. Case-case ORs of tumor characteristics by menopausal status among subjects ≥40 years of age in the Carolina Breast Cancer Study phases I–III. (DOC 88 kb) Additional file 3: Table S3. Case-control ORs of breast cancer risk factors by menopausal status among women ≥40 years of age, Carolina Breast Cancer Study phases I and II (*n* = 2185). (DOC 83 kb)
