# Supplementary material for: Breast cancer biologic and etiologic heterogeneity by young age and menopausal status in the Carolina Breast Cancer Study: a case-control study
Source: Breast Cancer Res. 2016 Aug 4;18:79. doi: 10.1186/s13058-016-0736-y (PMC4972943; doi:10.1186/s13058-016-0736-y)
Supplement: Additional file 1: Table S1. — Characteristics of control subjects by age and race in the Carolina Breast Cancer Study phases I and II (n = 2022). (DOC 61 kb) [file 13058_2016_736_MOESM1_ESM.doc]

Supplemental Table 1. Characteristics of controls by age and race in the Carolina Breast Cancer Study, Phases I-II (N=2,022).

|  | African American (N=788) | |  | Non-African American (N=1,234) | |
| --- | --- | --- | --- | --- | --- |
|  | <40 years | ≥40 years |  | <40 years | ≥40 years |
| Risk factor | N (%) | N (%) |  | N (%) | N (%) |
| Mean age (±standard deviation) | 35.0 (±3.0) | 54.3 (±9.8) |  | 35.6 (±3.4) | 55.1 (±10.0) |
| BMI |  |  |  |  |  |
| <25 (normal/under) | 29 (27.9) | 128 (19.2) |  | 68 (53.5) | 524 (47.9) |
| 25-29.9 (overweight) | 29 (27.9) | 203 (30.4) |  | 31 (24.4) | 338 (30.9) |
| ≥30 (obese) | 46 (44.2) | 337 (50.5) |  | 28 (22.1) | 233 (21.3) |
| *Missing* |  | 16 |  |  | 12 |
| WHR |  |  |  |  |  |
| <0.77 | 35 (35.0) | 104 (15.4) |  | 57 (46.0) | 419 (38.3) |
| 0.77-0.83 | 29 (29.0) | 213 (31.6) |  | 42 (33.9) | 362 (33.1) |
| ≥0.84 | 36 (36.0) | 358 (53.0) |  | 25 (20.2) | 313 (28.6) |
| *Missing* | 4 | 9 |  | 3 | 13 |
| Parity |  |  |  |  |  |
| Nulliparous | 23 (22.1) | 63 (9.2) |  | 27 (21.3) | 117 (10.6) |
| 1-2 births | 54 (51.9) | 278 (40.6) |  | 84 (66.1) | 597 (53.9) |
| ≥3 births | 27 (26.0) | 343 (50.2) |  | 16 (12.6) | 393 (35.5) |
| History of breastfeeding*^a^* |  |  |  |  |  |
| Never | 56 (69.1) | 365 (59.2) |  | 27 (27.0) | 541 (54.7) |
| Ever | 25 (30.9) | 252 (40.8) |  | 73 (73.0) | 448 (45.3) |
| Lifetime breastfeeding duration*^a^* |  |  |  |  |  |
| Never | 58 (71.6) | 368 (59.7) |  | 34 (34.0) | 557 (56.4) |
| >0-3 months | 9 (11.1) | 70 (11.4) |  | 18 (18.0) | 155 (15.7) |
| ≥4 months | 14 (17.3) | 178 (28.9) |  | 48 (48.0) | 275 (27.9) |
| *Missing* |  | 1 |  |  | 2 |
| Time since last term pregnancy*^a^* |  |  |  |  |  |
| <10 years | 55 (67.9) | 29 (4.7) |  | 69 (69.0) | 53 (5.4) |
| 10-19 years | 24 (29.6) | 160 (26.0) |  | 29 (29.0) | 237 (24.0) |
| ≥20 years | 2 (2.5) | 427 (69.3) |  | 2 (2.0) | 699 (70.7) |
| *Missing* |  | 1 |  |  |  |
| Age at first live birth*^a^* |  |  |  |  |  |
| <26 years | 58 (71.6) | 526 (85.3) |  | 53 (53.0) | 709 (71.7) |
| ≥26 years | 23 (28.4) | 91 (14.7) |  | 47 (47.0) | 280 (28.3) |
| Age at last live birth*^a^* |  |  |  |  |  |
| <30 years | 55 (67.9) | 381 (61.9) |  | 54 (54.0) | 595 (60.2) |
| ≥30 years | 26 (32.1) | 235 (38.1) |  | 46 (46.0) | 394 (39.8) |
| *Missing* |  | 1 |  |  |  |
| Age at menarche |  |  |  |  |  |
| <13 years | 56 (53.9) | 321 (47.1) |  | 61 (48.4) | 504 (45.7) |
| ≥13 years | 48 (46.2) | 360 (52.9) |  | 65 (51.6) | 599 (54.3) |
| *Missing* |  | 3 |  | 1 | 4 |
| Oral contraceptive use |  |  |  |  |  |
| Never | 19 (18.3) | 314 (46.1) |  | 15 (11.8) | 380 (34.6) |
| Ever | 85 (81.7) | 367 (53.9) |  | 112 (88.2) | 717 (65.4) |
| *Missing* |  | 3 |  |  | 10 |

*^a^*Among parous women
