# Supplementary material for: Breast cancer biologic and etiologic heterogeneity by young age and menopausal status in the Carolina Breast Cancer Study: a case-control study
Source: Breast Cancer Res. 2016 Aug 4;18:79. doi: 10.1186/s13058-016-0736-y (PMC4972943; doi:10.1186/s13058-016-0736-y)
Supplement: Additional file 2: Table S2. — Case-case ORs of tumor characteristics by menopausal status among subjects ≥40 years of age in the Carolina Breast Cancer Study phases I–III. (DOC 88 kb) [file 13058_2016_736_MOESM2_ESM.doc]

Supplemental Table 2. Case-case ORs of tumor characteristics by menopausal status among cases ≥40 years of age in the Carolina Breast Cancer Study, Phases I-III.

|  | All cases ≥40 years of age (N=4,601) | | | 40-49 years of age (N=1,930)*^d^* | | | 40-59 years of age (N=3,100)*^d^* | | |
| --- | --- | --- | --- | --- | --- | --- | --- | --- | --- |
|  | Postmenopausal  (ref) | Premenopausal | | Postmenopausal  (ref) | Premenopausal | | Postmenopausal  (ref) | Premenopausal | |
| Tumor characteristic | N (%) | N (%) | OR (95% CI) | N (%) | N (%) | OR (95% CI) | N (%) | N (%) | OR (95% CI) |
| Stage |  |  |  |  |  |  |  |  |  |
| In situ | 343 (12.3) | 125 (7.5) | 0.74 (0.59, 0.94) | 29 (8.0) | 106 (7.0) | 0.83 (0.52, 1.30) | 150 (11.4) | 125 (7.5) | 0.73 (0.56, 0.96) |
| Stage 1 | 1,170 (41.9) | 573 (34.5) | 1.0 | 116 (31.0) | 514 (34.0) | 1.0 | 505 (37.5) | 573 (34.6) | 1.0 |
| Stage 2 | 926 (33.2) | 687 (41.4) | 1.52 (1.32, 1.74) | 150 (41.2) | 636 (42.1) | 0.96 (0.73, 1.25) | 483 (35.9) | 687 (41.4) | 1.25 (1.06, 1.48) |
| Stage 3 | 279 (10.0) | 220 (13.3) | 1.61 (1.31, 1.97) | 58 (15.9) | 205 (13.6) | 0.80 (0.56, 1.14) | 164 (12.2) | 218 (13.2) | 1.17 (0.93, 1.48) |
| Stage 4 | 75 (2.7) | 55 (3.3) | 1.50 (1.04, 2.15) | 11 (3.0) | 50 (3.3) | 1.03 (0.52, 2.03) | 45 (3.3) | 55 (3.3) | 1.08 (0.71, 1.63) |
| *Missing*^c^ | 97 | 51 |  | 10 | 45 |  | 44 | 51 |  |
| Subtype*^a^* |  |  |  |  |  |  |  |  |  |
| Luminal | 539 (69.6) | 283 (65.2) | 1.0 | 57 (56.4) | 260 (64.5) | 1.0 | 243 (64.1) | 283 (65.2) | 1.0 |
| Basal-like | 97 (12.5) | 73 (16.8) | 1.43 (1.02, 2.00) | 21 (20.8) | 68 (16.9) | 0.71 (0.40,1.25) | 62 (16.4) | 73 (16.8) | 1.01 (0.69, 1.48) |
| HER2 | 67 (8.7) | 28 (6.5) | 0.80 (0.50, 1.27) | 13 (12.9) | 26 (6.5) | 0.44 (0.21, 0.91) | 40 (10.6) | 28 (6.5) | 0.60 (0.36, 1.00) |
| Unclassified | 71 (9.2) | 50 (11.5) | 1.34 (0.91, 1.98) | 10 (9.9) | 49 (12.2) | 1.07 (0.51, 2.25) | 34 (9.0) | 50 (11.5) | 1.29 (0.79, 2.02) |
| *Missing* | 487 | 286 |  | 55 | 257 |  | 209 | 286 |  |
| ER status |  |  |  |  |  |  |  |  |  |
| Negative | 777 (29.8) | 515 (33.4) | 1.0 | 136 (39.9) | 476 (33.9) | 1.0 | 447 (35.6) | 515 (33.4) | 1.0 |
| Positive | 1,830 (70.2) | 1,028 (66.6) | 0.85 (0.74, 0.97) | 205 (60.1) | 927 (66.1) | 1.29 (1.01, 1.65) | 807 (64.4) | 1,026 (66.6) | 1.10 (0.94, 1.29) |
| *Missing*^c^ | 283 | 168 |  | 33 | 153 |  | 137 | 168 |  |
| PR status |  |  |  |  |  |  |  |  |  |
| Negative | 958 (42.6) | 544 (37.6) | 1.0 | 159 (50.0) | 502 (37.9) | 1.0 | 548 (49.0) | 544 (37.6) | 1.0 |
| Positive | 1,293 (57.4) | 903 (62.4) | 1.23 (1.07, 1.41) | 159 (50.0) | 821 (62.1) | 1.64 (1.28, 2.09) | 570 (51.0) | 902 (62.4) | 1.59 (1.36, 1.87) |
| *Missing*^c^ | 639 | 264 |  | 56 | 233 |  | 273 | 263 |  |
| HER2 status*^a^* |  |  |  |  |  |  |  |  |  |
| Negative | 790 (82.8) | 447 (82.3) | 1.0 | 92 (79.3) | 418 (82.8) | 1.0 | 360 (81.3) | 447 (82.3) | 1.0 |
| Positive | 164 (17.2) | 96 (17.7) | 1.04 (0.78, 1.37) | 24 (20.7) | 87 (17.2) | 0.80 (0.48, 1.32) | 83 (18.7) | 96 (17.7) | 0.93 (0.67, 1.29) |
| *Missing* | 307 | 177 |  | 40 | 155 |  | 145 | 177 |  |
| Histologic grade*^b^* |  |  |  |  |  |  |  |  |  |
| Well/moderate differentiation | 529 (30.9) | 268 (25.6) | 1.0 | 57 (23.4) | 241 (25.1) | 1.0 | 210 (25.6) | 267 (25.6) | 1.0 |
| Poor differentiation | 1,183 (69.1) | 779 (74.4) | 1.30 (1.09, 1.55) | 187 (76.6) | 719 (74.9) | 0.91 (0.65, 1.27) | 611 (74.4) | 778 (74.4) | 1.00 (0.81, 1.24) |
| *Missing*^c^ | 326 | 238 |  | 42 | 215 |  | 167 | 238 |  |
| Nuclear grade*^b^* |  |  |  |  |  |  |  |  |  |
| Slight/moderate pleomorphism | 1,041 (59.9) | 557 (52.2) | 1.0 | 110 (44.2) | 503 (51.4) | 1.0 | 301 (51.2) | 366 (49.1) | 1.0 |
| Marked pleomorphism | 696 (40.1) | 511 (47.8) | 1.37 (1.18, 1.60) | 139 (55.8) | 475 (48.6) | 0.75 (0.57, 0.99) | 287 (48.8) | 379 (50.9) | 0.96 (0.80, 1.16) |
| *Missing*^c^ | 301 | 217 |  | 37 | 197 |  | 402 | 536 |  |
| Node status |  |  |  |  |  |  |  |  |  |
| Negative | 1,616 (65.1) | 907 (58.4) | 1.0 | 185 (54.7) | 825 (58.0) | 1.0 | 739 (60.8) | 907 (58.5) | 1.0 |
| Positive | 868 (34.9) | 645 (41.6) | 1.32 (1.16, 1.51) | 153 (45.3) | 597 (42.0) | 0.88 (0.69, 1.11) | 476 (39.2) | 643 (41.5) | 1.10 (0.94, 1.28) |
| *Missing*^c^ | 406 | 159 |  | 36 | 134 |  | 176 | 159 |  |
| Tumor size |  |  |  |  |  |  |  |  |  |
| ≤2 cm  >2 cm | 1,430 (58.7) | 749 (48.8) | 1.0 | 155 (47.4) | 680 (48.4) | 1.0 | 626 (52.9) | 749 (48.9) | 1.0 |
|  | 1,006 (41.3) | 786 (51.2) | 1.49 (1.31, 1.70) | 172 (52.6) | 724 (51.6) | 0.96 (0.75, 1.22) | 557 (47.1) | 784 (51.1) | 1.18 (1.01, 1.37) |
| *Missing*^c^ | 454 | 176 |  | 47 | 152 |  | 208 | 176 |  |

*^a^*Excludes Phase 3 cases

*^b^*Excludes Phase 2 cases

*^c^*Missing data due to ongoing data collection for Phase III cases.

*^d^*Sensitivity analyses with restricted age ranges to address residual confounding by age.
