# Supplementary material for: Breast cancer biologic and etiologic heterogeneity by young age and menopausal status in the Carolina Breast Cancer Study: a case-control study
Source: Breast Cancer Res. 2016 Aug 4;18:79. doi: 10.1186/s13058-016-0736-y (PMC4972943; doi:10.1186/s13058-016-0736-y)
Supplement: Additional file 3: Table S3. — Case-control ORs of breast cancer risk factors by menopausal status among women ≥40 years of age, Carolina Breast Cancer Study phases I and II (n = 2185). (DOC 83 kb) [file 13058_2016_736_MOESM3_ESM.doc]

Supplemental Table 3. Case-control ORs of breast cancer risk factors by menopausal status among women ≥40 years of age, Carolina Breast Cancer Study Phases I-II (N=2,185).

|  | Premenopausal | | |  | Postmenopausal | | |  | Test for heterogeneity |
| --- | --- | --- | --- | --- | --- | --- | --- | --- | --- |
| Risk factor | Controls N (%) | Cases N (%) | OR (95% CI)*^a^* |  | Controls N (%) | Cases N (%) | OR (95% CI)*^a^* |  | Χ^2^, degrees of freedom (*p*)*^b^* |
| BMI (kg/m^2^  ) |  |  |  |  |  |  |  |  |  |
| <25.0 | 255 (40.1) | 316 (44.3) | 1.0 |  | 185 (33.8) | 216 (37.2) | 1.0 |  | 2.58, 2 (0.3) |
| 25-29.9 | 184 (28.9) | 181 (25.4) | 0.86 (0.65, 1.14) |  | 168 (30.7) | 179 (30.9) | 0.83 (0.61, 1.13) |  |  |
| ≥30.0 | 197 (31.0) | 217 (30.4) | 0.98 (0.74, 1.29) |  | 195 (35.6) | 185 (31.9) | 0.63 (0.46, 0.87) |  |  |
| *Missing* | 12 | 6 |  |  | 5 | 8 |  |  |  |
| WHR |  |  |  |  |  |  |  |  |  |
| <0.77 | 235 (36.8) | 231 (32.4) | 1.0 |  | 159 (28.9) | 155 (26.8) | 1.0 |  | 0.83, 2 (0.7) |
| 0.77-0.83 | 209 (32.8) | 245 (34.4) | 1.42 (1.08, 1.86) |  | 175 (31.8) | 179 (31.0) | 1.09 (0.79, 1.51) |  |  |
| ≥0.84 | 194 (30.4) | 237 (33.2) | 1.58 (1.18, 2.12) |  | 216 (39.3) | 244 (42.2) | 1.15 (0.84, 1.58) |  |  |
| *Missing* | 10 | 7 |  |  | 3 | 10 |  |  |  |
| Parity |  |  |  |  |  |  |  |  |  |
| Nulliparous | 75 (11.6) | 106 (14.7) | 1.0 |  | 54 (9.8) | 81 (13.8) | 1.0 |  | 0.28, 2 (0.9) |
| 1-2 births | 383 (59.1) | 420 (58.3) | 0.80 (0.57, 1.13) |  | 270 (48.8) | 288 (49.0) | 0.69 (0.47, 1.04) |  |  |
| ≥3 births | 190 (29.3) | 194 (26.9) | 0.79 (0.54, 1.16) |  | 229 (41.4) | 219 (37.2) | 0.63 (0.42, 0.96) |  |  |
| *Missing* |  |  |  |  |  |  |  |  |  |
| History of breastfeeding*^c^* |  |  |  |  |  |  |  |  |  |
| Never | 343 (60.2) | 370 (60.6) | 1.0 |  | 328 (65.7) | 317 (62.8) | 1.0 |  | 0.56, 1 (0.5) |
| Ever | 227 (39.8) | 241 (39.4) | 0.90 (0.70, 1.16) |  | 171 (34.3) | 188 (37.2) | 1.09 (0.83, 1.45) |  |  |
| *Missing* |  |  |  |  |  |  |  |  |  |
| Lifetime breastfeeding duration*^c^* |  |  |  |  |  |  |  |  |  |
| Never | 346 (60.7) | 378 (61.9) | 1.0 |  | 333 (66.7) | 321 (63.6) | 1.0 |  | 1.28, 2 (0.5) |
| >0-3 months | 69 (12.1) | 65 (10.6) | 0.86 (0.58, 1.25) |  | 58 (11.6) | 59 (11.7) | 0.96 (0.63, 1.46) |  |  |
| ≥4 months | 155 (27.2) | 168 (27.5) | 0.87 (0.65, 1.15) |  | 108 (21.6) | 125 (24.8) | 1.16 (0.85, 1.60) |  |  |
| *Missing* |  |  |  |  |  |  |  |  |  |
| Time since last term pregnancy*^c^* |  |  |  |  |  |  |  |  |  |
| <10 years | 76 (13.3) | 108 (17.7) | 1.0 |  | 6 (1.2) | 5 (1.0) | 1.0 |  | 4.46, 2 (0.1) |
| 10-19 years | 302 (53.0) | 283 (46.3) | 0.71 (0.50, 1.01) |  | 95 (19.0) | 112 (22.2) | 1.05 (0.31, 3.60) |  |  |
| ≥20 years | 192 (33.7) | 220 (36.0) | 0.96 (0.64, 1.43) |  | 398 (79.8) | 388 (76.8) | 0.83 (0.24, 2.82) |  |  |
| *Missing* |  |  |  |  |  |  |  |  |  |
| Age at first live birth*^c^* |  |  |  |  |  |  |  |  |  |
| <26 years | 386 (67.7) | 414 (67.8) | 1.0 |  | 408 (81.8) | 388 (76.8) | 1.0 |  | 1.06, 1 (0.3) |
| ≥26 years | 184 (32.3) | 197 (32.2) | 0.93 (0.71, 1.20) |  | 91 (18.2) | 117 (23.2) | 1.26 (0.90, 1.74) |  |  |
| *Missing* |  |  |  |  |  |  |  |  |  |
| Age at last live birth*^c^* |  |  |  |  |  |  |  |  |  |
| <30 years | 344 (60.4) | 362 (59.2) | 1.0 |  | 347 (69.5) | 312 (61.8) | 1.0 |  | 3.19, 1 (0.2) |
| ≥30 years | 226 (35.6) | 249 (40.8) | 0.99 (0.78, 1.26) |  | 152 (30.5) | 193 (38.2) | 1.40 (1.07, 1.85) |  |  |
| *Missing* |  |  |  |  |  |  |  |  |  |
| Age at menarche |  |  |  |  |  |  |  |  |  |
| <13 years | 322 (49.7) | 393 (54.6) | 1.0 |  | 261 (47.5) | 326 (55.6) | 1.0 |  | 1.11, 1 (0.3) |
| ≥13 years | 326 (50.3) | 327 (45.4) | 0.82 (0.66, 1.03) |  | 289 (52.5) | 260 (44.4) | 0.69 (0.54, 0.89) |  |  |
| *Missing* |  |  |  |  | 3 | 2 |  |  |  |
| Oral contraceptive use |  |  |  |  |  |  |  |  |  |
| Never | 111 (17.2) | 125 (17.4) | 1.0 |  | 151 (27.5) | 159 (27.2) | 1.0 |  | 0.02, 1 (0.9) |
| Ever | 534 (82.8) | 593 (82.6) | 0.93 (0.69, 1.24) |  | 399 (72.5) | 426 (72.8) | 1.03 (0.78, 1.37) |  |  |
| *Missing* | 3 | 2 |  |  | 3 | 3 |  |  |  |

*^a^*Adjusted for matching factors race and age

*^b^*Likelihood ratio tests assessed menopause-related heterogeneity in risk factor associations by comparing the estimated log-likelihood of adjusted models to that of the adjusted model including a multiplicative interaction term for menopausal status and the corresponding risk factor (e.g., BMI*menopausal status). Statistically significant heterogeneity by menopausal status was defined with α=0.1.

*^c^*Among parous women
